# The Ews-ERG Fusion Protein Can Initiate Neoplasia from Lineage-Committed Haematopoietic Cells

**DOI:** 10.1371/journal.pbio.0030242

**Published:** 2005-06-28

**Authors:** Rosalind Codrington, Richard Pannell, Alan Forster, Lesley F Drynan, Angelika Daser, Nati Lobato, Markus Metzler, Terence H Rabbitts

**Affiliations:** **1**MRC Laboratory of Molecular Biology, Cambridge, United Kingdom; Institute for Cancer ResearchUnited Kingdom

## Abstract

The EWS-ERG fusion protein is found in human sarcomas with the chromosomal translocation t(21;22)(q22;q12), where the translocation is considered to be an initiating event in sarcoma formation within uncommitted mesenchymal cells, probably long-lived progenitors capable of self renewal. The fusion protein may not therefore have an oncogenic capability beyond these progenitors. To assess whether EWS-ERG can be a tumour initiator in cells other than mesenchymal cells, we have analysed Ews-ERG fusion protein function in a cellular environment not typical of that found in human cancers, namely, committed lymphoid cells. We have used *Ews-ERG* invertor mice having an inverted *ERG* cDNA cassette flanked by *loxP* sites knocked in the *Ews* intron 8, crossed with mice expressing Cre recombinase under the control of the *Rag1* gene to give conditional, lymphoid-specific expression of the fusion protein. Clonal T cell neoplasias arose in these mice. This conditional *Ews* gene fusion model of tumourigenesis shows that Ews-ERG can cause haematopoietic tumours and the precursor cells are committed cells. Thus, Ews-ERG can function in cells that do not have to be pluripotent progenitors or mesenchymal cells.

## Abstract 2

Using a mouse model, these authors study the potential of a known cancer-related gene to cause tumors in cells committed to different lineages.

## Introduction

Chromosomal translocations are characteristic of many human cancers, and are especially well studied in leukaemias, lymphomas, and sarcomas [1,2]. Epithelial tumours also carry chromosomal translocations, but while those in leukaemias, lymphomas, and sarcomas tend to be reciprocal translocations, those in carcinomas are often non-reciprocal translocations [3]. The main outcomes of the reciprocal translocations are either forced oncogene expression, as found in lymphoid malignancies, or gene fusion, found in both leukaemias and sarcomas [1,2]. Gene fusion occurs when the translocation breakpoints are within the introns of genes, such that the two translocated chromosomes have new exon organisation, leading to the formation of chimaeric mRNA species and, in turn, chimaeric proteins.

Some translocations must function within stem cells to initiate disease while others function in both stem cells and more committed cells to provide related or distinct functions (reviewed in [4]). Different tumour phenotypes can result from specific versions of related fusion proteins, for instance, the Philadelphia t(9;22) translocation (which yields the *BCR-ABL* [breakpoint cluster region gene–Abelson leukaemia oncogene] fusion) occurs in both myeloid- and lymphoid-lineage tumours, dictated by the position of the translocation junction within the *BCR* gene [5]. The biological consequences of the *BCR-ABL* fusion have been studied in relation to stem cell properties [6–8]. These studies show that the BCR-ABL fusion protein does not confer self-renewal potential on committed myeloid progenitors, whereas other translocation fusion proteins (e.g., MOZ-TIF2) do [7]. The *MLL* (mixed lineage leukaemia) gene has more than 30 known fusion partners [9,10], and retroviral transduction experiments with *MLL-ENL* fusion showed that the multipotent myeloid progenitors and committed myeloid progenitors could respond to an MLL fusion protein to become leukaemic, suggesting the existence of cancer stem cells distinct from multipotent stem cells of the tissue of origin [11]. While some translocations have these dual properties, others only function in committed cells such as those translocations mediated by the RAG-VDJ (recombinase activating gene and variable, joining, diversity regions) recombinase of the lymphoid lineage [1]. Thus, naturally occurring translocations, such as that of *LMO2* *(LIM domain only 2)* in T cell acute leukaemia, only function in committed cells.

Such issues are not confined to haematopoietic malignancies. The *EWS-FUS* family, typically involved in sarcoma aetiology, exhibits characteristics of tumour initiators [12–15]. The *EWS* gene, at Chromosome 22 band q12, was first identified in Ewing's sarcoma by its association with *FLI1* and *ATF1* [16,17] and subsequently found in subsets of Ewing's sarcoma with either *ERG* or *ETV1*, and *E1AF* and *FEV* [17–21]. In addition, the *EWS* gene is involved in other sarcomas and with yet different partners, such as *WT1* (Wilms tumour 1 gene) in desmoplastic small round cell sarcoma [22] or *CHN* in myxoid chondrosarcoma [23]. A further ramification of this infidelity is that the *FUS* gene, first identified by its fusion with the *CHOP* gene in t(12;16) of malignant myxoid liposarcoma and related to the *EWS* gene [24,25], has also been found in Ewing's sarcoma fused with the *ERG* gene, but also a similar *FUS-ERG* fusion has been described in some acute myeloid leukaemias [26–28]. Finally, the *EWS* gene can also be involved with the *CHOP* gene by chromosomal translocation in malignant myxoid liposarcoma [29], analogous to *FUS-CHOP*. The chromosomal translocations in Ewing's and other sarcomas are considered as primary initiating events. Thus the EWS fusions function in the cancer precursor cell and are required throughout tumour formation until the emergence of the overt cancer, because the chromosomal translocation is present in the cancer at the time of presentation. The fusion protein may therefore be instructive by imparting a phenotype on the cell by affecting a specific differentiation programme, which must be part of the genetic make-up of the cancer stem cell, since the chromosomal translocation is an initiating and persistent feature. The pre-existing chromosomal translocation creates a cellular environment allowing secondary mutations to arise in progeny cells, resulting in overt cancer. Conditional mouse models of the *EWS*- and *FUS*-associated chromosomal translocations should give insights into how these fusions influence the malignant phenotype. We have analysed whether EWS fusions function only in the domain of mesenchymal progenitors and whether they function in other cell lineages, specifically within committed cells, as has been demonstrated for MLL fusions [11].

**Figure 1:**
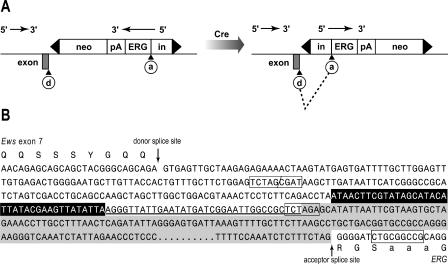
Strategy for Generating *Ews-ERG* Invertor Gene by Homologous Recombination (A) The method for making invertor mice is described in detail elsewhere [35]. In summary, an invertor cassette comprising a short intronic region with an acceptor splice site, human *ERG* cDNA sequence (shown in Figure S1), a polyA site, and the *MC1neopA* gene all flanked by *loxP* sites (depicted by black triangles) was knocked in, using homologous recombination, into *Ews* intron 8. The transcription orientation of the targeted *ERG* cDNA invertor cassette was opposite from that of the endogenous *Ews* gene after initial homologous recombination. Germ line carrier mice of this targeted allele were crossed with *Cre*-expressing mouse strains [36], and, as indicated in the right hand side of the diagram, the invertor cassette is turned around to create a transcription orientation identical with that of *Ews*. Thus, after transcription, a pre-mRNA is made with the donor splice site of *Ews* exon 7 adjacent to the acceptor site of the invertor cassette, allowing post-transcriptional fusion of *Ews* with *ERG* in an analogous format to that found in human sarcomas with t(21;22). (B) Genomic sequence adjacent to the *Ews* exon 7 and the *ERG* invertor cassette (the derived amino acid sequence is shown in the single letter code) obtained from DNA of ES cells and of thymus after Cre-mediated inversion. The *Ews* intron 7/8 donor splice site is indicated by an arrow. The boxed sequence ( TCTAG/ CGAT) corresponds to the ligation of filled-in XbaI (*Ews* genomic) and ClaI (*ERG* invertor cassette) sites (for detailed description see [35]). The boxed XbaI site ( TCTAGA) corresponds to the position of cloning of the mouse *Af4* intronic sequence (shaded) in the *ERG* invertor cassette. On the 3′ side of the fused XbaI-ClaI sites, there is a *loxP* site originating from the *ERG* invertor cassette (see Figure S1) followed by the *Af4* intron 4 (shaded) upstream of the *ERG* cDNA sequence. The *Af4* acceptor splice site is indicated by an arrow, and is followed by the *ERG* cDNA sequence. A NotI site used for cloning the amplified *ERG* sequence is boxed (see also Figure S1) (note this sequence is non-contiguous and the dots represent a gap; the full sequence appears in [35]).

We have developed mouse models mimicking chromosomal translocations by employing homologous recombination in embryonic stem (ES) cells to create mutant alleles in mice. These methods were either the knock-in model, which involved fusing the *MLL* gene partner *AF9* into the mouse *Mll* gene, resulting in acute myeloid leukaemias in mice [30,31], or the translocator model [32,33] to give de novo chromosomal translocations, resulting in cell-specific leukaemias [34]. We have applied a new conditional chromosomal translocation model (designated the invertor model) [35] to the *EWS-ERG* gene fusion to gain insights into the functional significance of the fusion protein in tumourigenesis. Invertor mice are made by knocking a floxed cDNA cassette into the intron of a target gene downstream of the mouse exon equivalent to that usually involved in human chromosomal translocations [35]. The floxed cassette is introduced into the intron in a reverse transcriptional orientation and can be inverted by Cre recombinase to create a transcription unit capable of generating an appropriate fusion mRNA (see below and Figure 1A). We have sought to determine whether an Ews-ERG fusion can be oncogenic in a cell type not generally associated with human tumours (specifically in lymphoid cells) and also to determine whether Ews-ERG fusion can initiate leukaemia from committed cells. We found that clonal T cell lymphoma arose in the invertor model when Cre expression was controlled by the lymphocyte-specific *Rag1* gene, showing that Ews-ERG is leukaemogenic in lymphoid cells. Our results show that *Ews-ERG* can function as an oncogene in committed cells of mice and suggest that EWS-ERG is able to contribute to neoplasia in a variety of cellular contexts in vivo.

## Results

### Conditional Inversion of the *ERG* Gene and Fusion with *Ews*

The use of homologous recombination to generate oncogene fusions was established with the creation of the *Mll-AF9* mouse model [30]. We attempted a similar approach to make *Ews* fusions of the type found in specific human sarcomas and leukaemias (Figure S1 shows sequences of *Chop,* *ATF1,* and *Fli1* together with *AF9* sequences used for the knock-in targeting clones). While we were able to obtain homologous recombination knock-in clones either with a vector carrying only a transfer cassette or with the *Ews-AF9* control fusion, no clones were obtained with any of the *Ews* fusions equivalent to those naturally found in human cancers (Table S1). Furthermore, chimaeras could be made with the *Ews* cassette and the *Ews-AF9* knock-in (Table S1). We concluded that Ews fusions of the type naturally associated with human cancers could be lethal in ES cells, obviating the generation of targeted cells.

We have recently developed a new conditional knock-in method based on inversion of a *loxP*-flanked cDNA cassette by Cre recombinase [35]. This *Ews-ERG* invertor model is diagrammatically shown in Figure 1A. In outline, homologous recombination in mouse ES cells was used to introduce a floxed *ERG* cDNA cassette downstream of *Ews* exon 7. The cassette comprised a short intronic sequence, an acceptor splice site, *ERG* cDNA, a polyA site, and a neomycin gene (to select homologous recombinant ES cells), placed in the opposite transcription orientation to the *Ews* gene. Expression of Cre recombinase inverts the cassette, bringing the acceptor splice site into the correct orientation with the *Ews* exon 7 donor splice site, to allow post-transcriptional production of *Ews-ERG* fusion mRNA (Figure 1B shows the post-inversion genomic sequence at the junction of *Ews* and *ERG*). Targeted ES cells were injected into blastocysts, and these yielded normal numbers of chimaeras, which gave rise to heterozygous carriers of the *Ews-ERG* invertor allele (invertor mice). In these invertor mice, Ews-ERG protein can only be made after Cre-mediated inversion and thus can be cell-specifically dependent on Cre expression.

### The Ews-ERG Fusion Protein Causes T Cell Neoplasia in Invertor Mice

**Figure 2:**
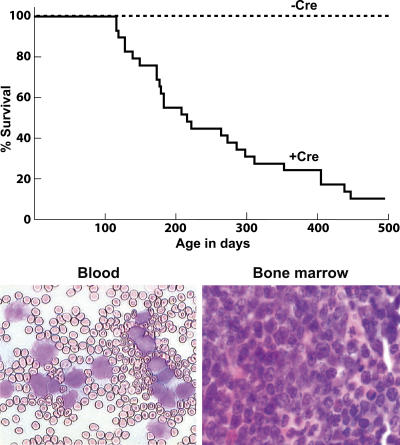
Incidence of Haematological Malignancy and Characteristics of Ews-ERG Invertor Mice Cohorts of 29 Ews-ERG; Rag1-Cre mice and 20 Ews-ERG control mice were monitored over a period of approximately 17 mo. Mice were culled and a post-mortem conducted when signs of ill health were observed. Leukaemia/lymphoma was established by various criteria (see Materials and Methods). Top panel shows the survival curve of Ews-ERG; Rag1-Cre (+Cre) or Ews-ERG (−Cre) mice as a function of time (days). Bottom panel shows the histology of blood and bone marrow from leukaemic mice (M21 and M20, respectively). Blood smears were stained with May-Grünwald-Giemsa stain and photographed at 400× magnification, whilst bone marrow was photographed at 1,000×.

We investigated the ability of Ews-ERG fusion protein to contribute to leukaemogenesis by causing the inversion of the *ERG*-containing cassette in lymphoid cells using *Rag1-Cre* knock-in mice [36]. Out of the cohort of 29 mice carrying both *Ews-ERG* and *Rag1-Cre* genes, 25 mice (86%) developed leukaemia associated with thymoma within 500 d, whereas the *Ews-ERG*-only cohort (20 mice) did not display neoplasia (Figure 2). Blood smears at the time of sacrifice of *Ews-ERG; Rag1-Cre* mice typically showed elevated numbers of large leukocytes, with lymphoid morphology of different maturities, including lymphoblasts (Figure 2). In addition, bone marrow sections showed high levels of infiltrating lymphoblasts (Figure 2). The diseased mice all had large thymomas, usually associated with splenomegaly (Table 1) and lymphadenopathy, and the histology of spleens showed either complete loss of normal architecture (loss of demarcated white and red pulp), partial loss, or normal architecture (Figure S2). Similarly, the level of infiltration of leukaemic lymphocytes into liver and kidney varied, with some having large amounts of perivascular deposits and others marginal levels. All mice had thymomas with homogeneous presence of large leukaemic cells. These characteristics, together with evidence of clonal *Tcrb* gene rearrangements and expression of TCR-associated CD8 and CD4 (see below), permit diagnosis of large cell anaplastic T cell lymphoma according to the Bethesda proposals for lymphoid tumours in mice [37].

**Table 1:**
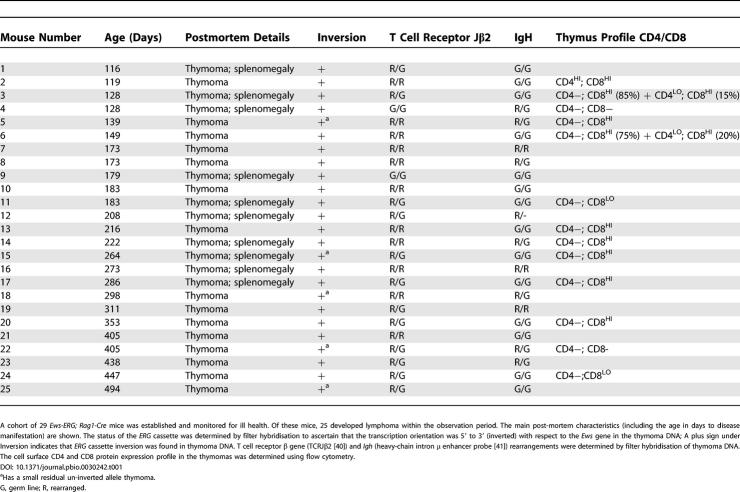
Tumour Characteristics of *Ews-ERG* Invertor Mice Expressing Cre Recombinase from the *Rag1* Gene

**Figure 3:**
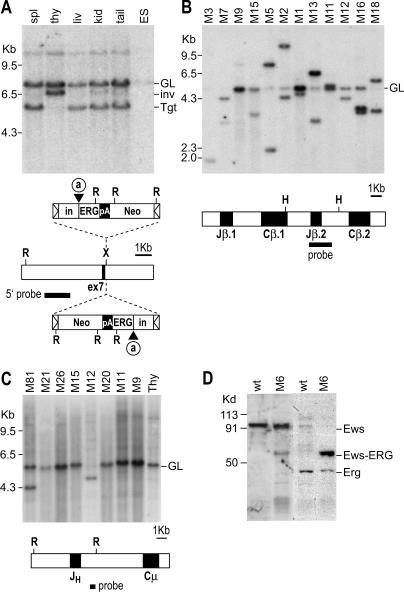
Clonality of T Cell Neoplasias in*Ews-ERG* Invertor Mice Genomic DNA was prepared from various tissues of invertor mice with thymomas. Genomic analysis was carried out using filter hybridisation to assess the presence of the inverted *ERG* cassette in the tumour cells (A), and to assess whether the lymphocytes involved in the tumours were clonal T (B) or B cells (C). (A) Inversion hybridisation autoradiograph. DNA was prepared from the thymoma and other tissues of M18, cleaved with EcoRI and hybridised to the 5′ *Ews* probe (which detects a 5-kb targeted *ERG* cassette fragment or a 6.5-kb Cre-inverted fragment). If the *ERG* cassette is inverted by Cre activity, the size of the EcoRI fragment increases from the initial targeted gene size, as indicated in the maps below the figure. The data shown are for DNA extracted from spleen (spl), thymus (thy), liver (liv), kidney (kid) or tail (ES cell DNA is used as a control). The Cre-mediated inverted band (∼6.5 kb) is evident in thymus DNA (thymoma). The summary of the data from the cohort of invertor mice is in Table 2. Note that despite extensive infiltration of spleen detected by histology, we cannot see the inverted band by Southern blot; this presumably reflects regional clustering of neoplastic cells in spleen. Restriction fragment sizes are represented as germ line (GL), inverted allele of *ERG* cassette (inv) and initial targeted *Ews* allele (Tgt). The organisation of the targeted *Ews* allele is indicated underneath. The hybridisation autoradiograph shows the location of *Ews* exon 7 and the initial targeted orientation (bottom) or inverted orientation of *ERG* invertor cassette after Cre-mediated recombination (top). a, acceptor splice site. (B) Autoradiograph showing rearrangement of T cell receptor β locus. A T cell receptor Jβ2 probe [40] (diagrammatically shown below the autoradiograph) detects a 5-kb germ line HindIII Jβ2 band whilst V-D-Jβ2 joins in T cells result in new HindIII-sized bands depending on the nature of the rearrangement. Each of 12 thymoma DNAs that were compared showed one or two Jβ2 alleles rearranged, signifying that these tumours were clonal T cells. In some thymoma samples, there is almost complete absence of the germ line band, indicating that the thymuses of these mice are solely comprised of malignant clonal T cells. DNA sequence analysis of the V-D-J junctions of mice M2, M5, M13, and M18 showed functional V-D-J joins (see Table 2). (C) Autoradiograph showing rearrangement status of immunoglobulin heavy-chain genes. A Cμ intron probe [41] (diagrammatically shown below the autoradiograph) was used to hybridise a set of thymoma DNAs for the presence of *Igh* rearranged bands. Only two samples showed rearrangements. (D) Detection of Ews-Erg fusion protein in thymoma cells. Single cell suspensions were made of T cells from a normal thymus (wt) or from the thymoma of M6, protein fractionated on 4%–20% acrylamide gel, and transferred to nylon membranes. Specific proteins were detected with anti-Ews or anti-ERG antibody.

The Ews-ERG fusion appears crucial for malignancies in the invertor mice, as disease only arose in mice with both the *Ews-ERG* and *Rag1-Cre* genes (Figure 2) and comprised clonal tumours (Figure 3). The presence of *Ews-ERG* mRNA was confirmed in spleens of these mice, identical with that in *Ews-ERG* ES cells transfected with *Cre* expression plasmids (see Figure S1). In addition, the steady state orientation of the *loxP*-flanked *ERG* cassette was studied using filter hybridisation and we found that the *ERG* sequence had become inverted into the 5′ to 3′ orientation with respect to the *Ews* gene in each case of thymoma (Figure 3A; Table 1). The other tissues examined have little evidence of an inverted band. Finally, the presence of the Ews-ERG fusion protein was shown by Western blotting of thymoma proteins using antibodies binding to either Ews or ERG, which detected respectively the normal mouse Ews or Erg proteins and the Ews-ERG fusion molecule in thymoma T cells of an *Ews-ERG; Rag1-Cre* mouse (Figure 3D).

**Table 2:**
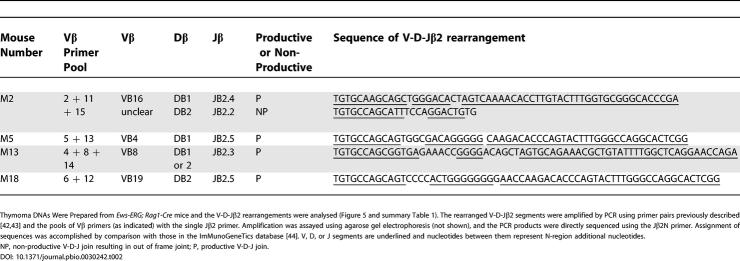
Sequences of Rearranged TCR Jβ Loci in *Ews-ERG* Thymoma DNA

Studies of progenitor gene expression have indicated the promiscuous expression of lymphoid markers precedes lineage commitment [38] and thus, the *Rag1-Cre* allele might be expressing in cells destined to become non-lymphoid. The *Rag1-Cre* knock-in allele was previously shown to be specific for lymphoid cells using a reporter assay dependent on Cre-mediated deletion of a *loxP*-flanked (floxed) segment of the *Lmo2* gene [36]. We have sustained these observations using an additional reporter assay, namely the *ROSA-loxSTOP-lacZ (ROSA26-R)* allele [39] where β-galactosidase (βgal) expression is activated by deletion of a *loxP-pA* site. Haematopoietic populations from mice carrying both *Rag1-Cre* and *ROSA26-R* alleles were stained with fluorescent antibodies binding to various surface markers, and flow cytometry was carried out for co-detection of antibody- and βgal-derived fluorescence (Figure S3). βgal signal was found in thymus and spleen (almost all cells), and in bone marrow, a population of βgal+ cells exist but did not co-express with CD34, Sca1, Ckit, or Ter119 (or Mac1, not shown). This means that the Rag1-Cre is restricted to lymphoid cells, as previously shown [36].

**Figure 4:**
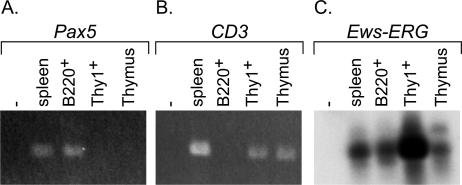
B and T Cells Express the Ews-ERG Fusion RNA A 96-d-old mouse with both *Ews-ERG* and *Rag1-Cre* alleles was used as a source of spleen and thymus cells. Single cell suspensions of spleen cells were labelled with anti-B220 or with anti-Thy1.2 and were purified using a MoFlo preparative flow cytometer. Estimated purities were achieved of greater than 95%. cDNA was prepared from RNA extracted from sorted cells or from aliquots of unsorted populations and RT-PCR (approximately 3,400 B220+ or 6,400 Thy1.2+ cell equivalents per PCR reaction) carried out with specific *Pax5* (A), *CD3* (B) or *Ews-ERG* (C) primers. PCR reaction products were fractionated on 1% agarose gels and either stained with ethidium bromide and photographed (A and B) or gel blotted and hybridised with an *Ews-ERG* probe (C)

The *Rag1-Cre* gene is expressed in both B and T cells, but only T cell tumours have arisen in the *Ews-ERG; Rag1-Cre* invertor line. The possible inversion of the *Ews-ERG* gene in B cells was investigated using RT-PCR analysis of expressed *Ews-ERG* fusion mRNA (Figure 4). RNA was prepared from whole spleen or thymus or from flow-sorted B220+ spleen cells (3,400 cells) or Thy1.2+ spleen cells (6,400 cells) and RT-PCR performed. *Pax5* and *CD3* primers [38] were used for specific detection of B cell and T cell transcripts, respectively. *Pax5* transcripts were detected in cDNA made from spleen and B220+ sorted cells, and *CD3* in the spleen, thymus and thy1.2+ sorted cells (Figure 4A and 4B). No evidence of *CD3* expression was found in the sorted B220+ cells, or of *Pax5* in the sorted Thy1.2+ cells, showing that the sorted cells were practically free of contaminating T or B cells, respectively. The presence of *Ews-ERG* fusion RNA was analysed with RT-PCR primers, yielding a product spanning the fusion junction that was detected with an internal junction probe. *Ews-ERG* RT-PCR product was detected in the unfractionated spleen and thymus sources, as well as in the purified, sorted B220+ and Thy1.2+ cells. Therefore, Cre-mediated inversion of the *Ews-ERG* gene occurs in both T and B cells.

### Ews-ERG Induces Malignancies of Mature T Cells

**Figure 5:**
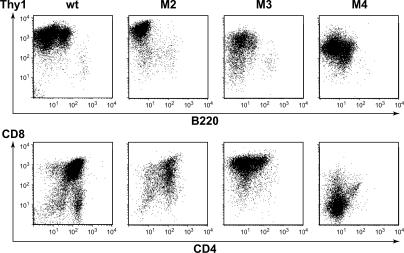
Analysis of Cell Surface Antigens of Thymomas from *Ews-ERG* Invertor Mice Using Flow Cytometry Thymus tissue was resected from mice with thymoma and FACS analysis performed to determine T cell phenotype (summarised in Table 1). The data in the figure show representative flow diagrams of three tumour-bearing mice (M2, M3, and M4) compared with a wild-type C57BL/6 control (wt), using anti-Thy1 (*y*-axis) plus anti-B220 (*x-*axis) antibodies or using anti-CD8 (*y*-axis) plus anti-CD4 (*x*-axis) antibodies. The three thymomas show a range of CD4 and CD8 co-expression phenotypes characterizing the thymomas generated in the *Ews-ERG; Rag1-Cre* invertor mice..

The presence of thymomas in all afflicted *Ews-ERG; Rag1-Cre* mice suggested that the haematological malignancy in these mice comprised T cells. This was confirmed by FACS analysis of T cell surface markers and by determination of T cell or B cell receptor gene rearrangement status. The majority of normal mouse thymic T cells express both the CD4 and CD8 surface markers (double positive [DP] cells), as well as the pan-T cell marker Thy1 (CD90) (wt in Figure 5). The situation with the thymomas of invertor mice varied from mainly CD4+CD8+ DP cells (e.g., M2 in Figure 5) to mainly CD8+ single positive (SP) cells (e.g., M3 in Figure 5) to mainly double negative cells (e.g., M4 in Figure 5). Thirteen of the cohort of 25 leukaemic invertor mice were analysed for their thymic T cell profile by FACS (summarized in Table 1). While we found either CD8+ SP or CD4+CD8+ DP thymomas, none showed a CD4+ SP phenotype. Two of the thymomas had a mixed phenotype comprising mainly CD8+ SP cells, with a sub-population of DP cells (M3 and M6 in Table 1).

The clonality of these T cell tumours was assessed by filter hybridisation of genomic thymoma DNA with a T cell receptor β probe *(Tcrb)* from the Jβ2 region [40] (diagrammatically shown in Figure 3B). The probe detects a 5-kb band in non-lymphoid DNA corresponding to the intact *Tcrb* gene; if V-D-J or D-J joins have occurred, new restriction fragments are created, giving rise to “rearranged” bands on the hybridisation autoradiograph. Figure 3B shows the rearrangement status of 12 of the *Ews-ERG* thymomas. All except one (M9) showed at least one rearranged *Jβ2* allele and several have two rearranged alleles (e.g., M18). Variable amounts of residual germ line allele were present. The *Tcrb* rearrangement status of the cohort of *Ews-ERG* leukaemic mice is summarized in Table 1. In addition, the status of the immunoglobulin locus was examined with a probe from the region between JH and Cμ segments [41] (see Figure 3C). Single *Igh* allele rearrangements were observed in about half of the cohort (Table 1), including ones in which FACS analysis and *Tcrb* clonality hybridisations showed the tumours to be clonal T cell malignancies. The *Igh* rearrangements are therefore likely to be abortive rearrangements.

Confirmation that the *Tcrb* rearrangements observed by hybridisations were due to productive V-D-J joins was made by sequence analysis of the *Tcrb* genes in four of the cases. PCR amplification from thymoma DNA was carried out with pools of Vβ primers and a Jβ2 reverse primer (primer sequences from [42,43]) and sequences identified with the ImMunoGeneTics database [44,45]. Table 2 shows the V-D-J junctional sequences indicating the presence of productive T cell receptor β genes. In all cases, N-region diversity is present between the V-D and D-J junctions. In the thymoma DNA from M2, a non-productive join has also occurred, in addition to the productive one, resulting in an out-of-frame joint.

## Discussion

### EWS-ERG Can Mediate Haematopoietic or Mesenchymal Tumourigenesis

Human cancers of mesenchymal origin involve the *EWS* gene with various partner genes, such as in the chromosomal translocation t(21;22)(q22;q12) where *EWS* fuses with *ERG* to encode a novel EWS-ERG fusion protein [18]. In addition, *EWS* gene fusions with various partners are described in clear cell sarcoma, desmoplastic small round cell tumours, chondrosarcoma, and myxoid liposarcoma, suggesting that EWS fusion proteins are functional for various cell types of mesenchymal origin and that restrictions in humans are dictated largely by the cells in which the chromosomal translocations occur. We tested the hypothesis of cell type specificity by assessing the possible universality of *EWS-ERG* as an oncogene. Our experimental system was designed to invoke conditional, cell-specific expression of the Ews-ERG fusion to analyse the target cell in which it can function. Consistent EWS-ERG occurrence in human sarcomas suggests that it may only function in mesenchymal cells, where it may function in initiation of the cancer as well as in maintenance of the cancer stem cell (i.e., cells within the tumour that have self-renewing capacity and maintain the tumour). Our results show that lymphomas arise when Ews-ERG is aberrantly expressed in the committed cells of the lymphoid lineage.

The *Ews-ERG* invertor allele was activated in lymphocytes, using the *Rag1-Cre* knock-in mice [36], to determine if lymphoid malignancies would arise. We found that invertor *Ews-ERG* mice develop clonal T cell malignancies. The T cells were of varying phenotypes but the majority of cases expressed CD8 on the cell surface and had productive V-D-Jβ rearrangements, indicative of mature T cells. Since Cre-mediated inversion through *loxP* sites recreates the *loxP* sequence, there is potential for the cassette to flip back and forth with continued Cre expression. This had no obvious deleterious effect on tumourigenesis in the *Ews-ERG* invertors as judged by various criteria (tumour penetrance, consistent T cell phenotype, and pathology). Thus, the invertor allele of transformed cells may be fixed in the correct orientation for synthesis of *Ews-ERG* mRNA when *Rag1-Cre* is no longer effective. On the other hand, the lack of CD4+ SP thymomas is difficult to encompass with this explanation, suggesting that other biological mechanisms may be involved in this bias.

The *Rag1-Cre* mouse line used constitutively expresses Cre in lymphoid cells [36], and the resulting Ews-ERG fusion causes T lymphocyte tumours in mice, which is not known in the spectrum of EWS-associated human cancers. On the other hand, FUS-ERG has been found in leukaemias, albeit myeloid type [26,46]. This implies that the EWS-FUS fusion proteins could be effective in a spectrum of cell types, broader than normally seen in human malignancies (i.e., sarcomas and myeloid leukaemias). In some cells, it may be that EWS-FUS fusions are lethal and thus those cells acquiring a translocation would die; in others, the fusion protein may be tolerated and thus may become tumours. In this respect, the absence of B cell tumours in the *Ews-ERG* invertors is of interest as both B and T cells undergo inversion of the *Ews-ERG* cassette (see Figure 4) because *Rag1-Cre* is expressed in both cell types [36] (see Figure S3). The absence of B cell tumours may reflect toxicity of the fusion protein for B cells although this seems unlikely given that we can detect the fusion mRNA in selected B220+ B lymphocytes (see Figure 4). Alternatively, it may mean that Ews-ERG does not function in B cells or that the development of B cell tumours is inhibited by the T cell malignancies, which may arise earlier and then dominate the lymphoid compartment. The ability of Ews-ERG to specifically cause B cell tumours could be evaluated by using B-cell-specific Cre-expressing mouse lines such as *CD19-Cre*.

### Ews-ERG Can Promote Tumourigenesis in Committed Cells

Our results show that the Ews-ERG fusion, normally restricted to sarcomas in humans, can initiate T lymphocyte tumours, if conditionally expressed in committed lymphoid cells. Leukaemogenesis in *Ews-ERG* invertor mice thus concurs with the hypothesis that EWS fusions can cause neoplasia arising in various cell types. Further, our data imply that the cellular context of *EWS*-associated chromosomal translocations in humans does not have to be a stem cell or even a multi-potent progenitor, as committed lymphocyte precursors expressing *Rag* recombinase genes are the leukaemic precursors in our invertor model. The same conclusions can be applied to *FUS*-associated chromosomal translocations that seem to be largely interchangeable with *EWS,* given the spectrum of cancers in which these are found and the relatedness of *FUS-* and *EWS*-coded proteins [24,25]. Thus, *EWS* or *FUS* chromosomal translocations probably arise more by virtue of accessibility of the genes to chromosomal translocations than by the precise cellular specificity of the resultant fusion proteins.

## Materials and Methods

### Generation of targeting constructs and homologous recombination

The *Ews* genomic targeting clone was constructed from γ phage DNA isolated from mouse 129 DNA [47]. The knock-in clones for potential *Ews* fusions with *AF9, Fli1, Chop,* and *ATF1* were made using a transfer vector pC2A-neo [35], and the sequences of these cDNA cassettes are given in Figure S1. Knock-in was achieved by homologous recombination as described [47]. The *ERG* inversion knock-in cassette [35] was prepared using cDNA made from human colon carcinoma *COLO320* mRNA (the sequence of the *ERG* segment, corresponding to the part of *ERG* found in *EWS-ERG* fusion [19] is shown Figure S1). A diagram of overall structure of the *ERG* inversion cassette is shown in Figure 1A, indicating the initial orientation of the *ERG* inversion cassette (3′ to 5′ with respect to *Ews*) and this was determined by restriction site mapping and genomic DNA sequencing. After identification of targeted ES cells, these were injected into C57BL/6 blastocysts, chimaeric mice were produced, and germ line transmission of the inversion knock-in allele was obtained. These mice were bred with mice expressing Cre from a *Rag1* knock-in [36]. Specificity of expression of the *Rag1-Cre* allele has previously been described [36] and was sustained using the ROSA26-R reporter mice [39] (see Figure S3).

### Molecular analyses

Genomic DNA was prepared from tissues using proteinase K digestion and phenol-chloroform extraction, and filter hybridisations were carried out with radiolabelled probes as described [48,49]. The probes used to detect antigen receptor gene rearrangements were a heavy-chain Ig μ intron enhancer probe [41] and a T cell receptor Jβ2 probe [40]. T cell receptor V-D-Jβ junction sequences were obtained by PCR amplification from thymoma DNA with pools of Vβ primers and a Jβ2 reverse primer (primer sequences from [42,43]), and the product was fractionated on agarose to determine the presence of a single amplified band. In turn, each band was eluted and the sequence obtained using the Jβ2N primer [43] and identified by comparison with the ImMunoGeneTics database [44,45].

RT-PCR was carried out on cDNA as described [34]. RNA was prepared using RNeasy (Quiagen, Valencia, California, United States) from cells that were labelled with FITC-conjugated antibodies and isolated by flow cytometry. Sequences have been described for *Pax5* and *CD3* RT-PCR primers [38], and the sequences of the *Ews-ERG* primers were 5′- CCACAGGATGGTAACAAGCCTGC-3′ *(Ews)* and 5′- CGAACTTGTAGGCGTAGC-3′ *(ERG)*. The hybridisation probe was a 303-bp fragment of *Ews-ERG* residing within the RT-PCR product.

### Analysis of leukaemia/lymphoma

A cohort of mice was established by inter-breeding the *Ews-ERG* invertor line with a *Rag1-Cre* line [36] to generate littermates with *Ews-ERG + Rag1-Cre* or *Ews-ERG* genotypes. Genotypes were determined using filter hybridisation of genomic DNA from a small tail biopsy. The health status of these mice was monitored, and if signs of ill health appeared, mice were sacrificed and a post-mortem was carried out. Tissue samples were removed for single cell preparation for determination of surface protein expression phenotype, for nucleic acid preparation, or for fixation in 10% formalin for histology. Blood smears were prepared and stained with May-Grünwald-Giemsa. For histology, fixed tissues were embedded in wax and 0.4-μ sections made, stained with haematoxylin and eosin after mounting on slides. FACS analysis was conducted using a FACSCalibur with fluorescent antibodies purchased from BD Biosciences (San Jose, California, United States). Data were analysed with CellQuest software (BD Biosciences). Western protein detection was carried out as described [36] using 18 μg of protein per lane and proteins fractionated on 4%–20% SDS-PAGE. The separated proteins were electro-transferred to PVDF nylon membranes (Millipore, Billerica, Massachusetts, United States) and specific proteins detected using anti-Ews antibody (Santa Cruz Biotechnology, Santa Cruz, California, United States; raised against the amino terminus of Ews) or anti-ERG antibody (Santa Cruz Biotechnology; raised against the carboxy terminus of ERG). Antibody bound to filter was detected using secondary antibodies and ECL as described by the manufacturer (Amersham Biosciences, Amersham, United Kingdom).

## Supporting Information

Figure S1 Sequence of Mouse *FliI* RT-PCR Fragment (111 KB PDF).

Figure S2 Histological Characteristics of Leukaemias in *Ews-ERG* Invertor Mice (6.4 MB PDF).

Figure S3 ROSA26-R-βgal Reporter Assay for Expression of *Rag1-Cre* Allele (354 KB PDF).

Table S1 Frequency of Targeted Clones and Chimaera Generation with *Ews* Knock-In ES Clone (23 KB DOC).

## Abbreviations

β-gal: β-galactosidase
DP: double positive
ES: embryonic stem cells
*ROSA26-R*: *ROSA-loxSTOP-lacZ*
SP: single positive
