# Supplementary material for: The Ews-ERG Fusion Protein Can Initiate Neoplasia from Lineage-Committed Haematopoietic Cells
Source: PLoS Biol. 2005 Jun 28;3(8):e242. doi: 10.1371/journal.pbio.0030242 (PMC1159166; doi:10.1371/journal.pbio.0030242)
Supplement: Supplementary file 1 [file pbio.0030242.sg001.pdf]

Supplemental Figure S1

A. Sequence of mouse *FliI* RT-PCR fragment

```
         10        20        30        40        50
gcggccgcAGACCCTTCTTATGACTCTGTCAGGAGAGGAGCATGGAACAA
cggccggcTCTGGGAAGAATACTGAGACAGTCCTCTCCTCGTACCTTGTT
 a  a  a  D  P  S  Y  D  S  V  R  R  G  A  W  N  N

         60        70        80        90       100
TAATATGAACTCTGGCCTCAACAAAAGTCCTCTCCTTGGAGGATCACAGA
ATTATACTTGAGACCGGAGTTGTTTTCAGGAGAGGAACCTCCTAGTGTCT
  N  M  N  S  G  L  N  K  S  P  L  L  G  G  S  Q

        110       120       130       140       150
CCATGGGCAAGAACACTGAGCAGCGGCCCCAGCCAGATCCTTATCAGATC
GGTACCCGTTCTTGTGACTCGTCGCCGGGGTCGGTCTAGGAATAGTCTAG
T  M  G  K  N  T  E  Q  R  P  Q  P  D  P  Y  Q  I

        160       170       180       190       200
CTGGGGCCAACCAGCAGCCGCCTAGCAAACCCTGGGAGTGGGCAGATCCA
GACCCCGGTTGGTCGTCGGCGGATCGTTTGGGACCCTCACCCGTCTAGGT
 L  G  P  T  S  S  R  L  A  N  P  G  S  G  Q  I  Q

        210       220       230       240       250
GCTGTGGCAGTTTCTCCTGGAACTACTGTCCGACAGCGCCAACGCCAGCT
CGACACCGTCAAAGAGGACCTTGATGACAGGCTGTCGCGGTTGCGGTCGA
  L  W  Q  F  L  L  E  L  L  S  D  S  A  N  A  S

        260       270       280       290       300
GTATCACCTGGGAGGGGACCAACGGGGAGTTCAAAATGACGGACCCTGAT
CATAGTGGACCCTCCCCTGGTTGCCCCTCAAGTTTTACTGCCTGGGACTA
C  I  T  W  E  G  T  N  G  E  F  K  M  T  D  P  D

        310       320       330       340       350
GAGGTGGCCAGGCGCTGGGGAGAGCGGAAGAGCAAGCCCAACATGAATTA
CTCCACCGGTCCGCGACCCCTCTCGCCTTCTCGTTCGGGTTGTACTTAAT
 E  V  A  R  R  W  G  E  R  K  S  K  P  N  M  N  Y

        360       370       380       390       400
TGACAAGCTGAGCCGGGCCCTCCGATACTACTATGACAAAAACATTATGA
ACTGTTCGACTCGGCCCGGGAGGCTATGATGATACTGTTTTTGTAATACT
  D  K  L  S  R  A  L  R  Y  Y  Y  D  K  N  I  M

        410       420       430       440       450
CCAAAGTGCATGGCAAAAGGTATGCCTACAAGTTTGACTTCCATGGCATT
GGTTTCACGTACCGTTTTCCATACGGATGTTCAAACTGAAGGTACCGTAA
T  K  V  H  G  K  R  Y  A  Y  K  F  D  F  H  G  I

        460       470       480       490       500
GCCCAGGCCCTGCAGCCACATCCAACAGAGACATCCATGTACAAGTATCC
CGGGTCCGGGACGTCGGTGTAGGTTGTCTCTGTAGGTACATGTTCATAGG
 A  Q  A  L  Q  P  H  P  T  E  T  S  M  Y  K  Y  P

        510       520       530       540       550
CTCTGATATCTCCTACATGCCTTCCTACCATGCCCATCAACAGAAGGTGA
GAGACTATAGAGGATGTACGGAAGGATGGTACGGGTAGTTGTCTTCCACT
  S  D  I  S  Y  M  P  S  Y  H  A  H  Q  Q  K  V
```

```
       560       570       580       590       600
ACTTTGTCCCGTCTCACCCATCCTCCATGCCTGTCACCTCCTCCAGCTTC
TGAAACAGGGCAGAGTGGGTAGGAGGTACGGACAGTGGAGGAGGTCGAAG
N  F  V  P  S  H  P  S  S  M  P  V  T  S  S  S  F

       610       620       630       640       650
TTTGGAGCAGCATCACAATACTGGACCTCCCCCACTGCTGGGATCTATCC
AAACCTCGTCGTAGTGTTATGACCTGGAGGGGGTGACGACCCTAGATAGG
 F  G  A  A  S  Q  Y  W  T  S  P  T  A  G  I  Y  P

       660       670       680       690       700
AAACCCCAGTGTCCCCCGCCATCCTAACACCCACGTGCCTTCACACTTAG
TTTGGGGTCACAGGGGGCGGTAGGATTGTGGGTGCACGGAAGTGTGAATC
  N  P  S  V  P  R  H  P  N  T  H  V  P  S  H  L

       710       720
GCAGCTACTACTAGcggccgc
CGTCGATGATGATCgccggcg
G  S  Y  Y  *
```

B. Sequence of human *AF9* RT-PCR fragment

```
        10        20        30        40        50
gcggccgcTCTTGAAGTGAAAAGTCCAATAAAGCAAAGCAAATCAGATAA
cgccggcgAGAACTTCACTTTTCAGGTTATTTCGTTTCGTTTAGTCTATT
 a  a  a  L  E  V  K  S  P  I  K  Q  S  K  S  D  K

        60        70        80        90       100
GCAAATAAAGAATGGTGAATGTGACAAGGCATACCTAGATGAACTGGTAG
CGTTTATTTCTTACCACTTACACTGTTCCGTATGGATCTACTTGACCATC
  Q  I  K  N  G  E  C  D  K  A  Y  L  D  E  L  V

       110       120       130       140       150
AGCTTCACAGAAGGTTAATGACATTGAGAGAAAGACACATTCTGCAGCAG
TCGAAGTGTCTTCCAATTACTGTAACTCTCTTTCTGTGTAAGACGTCGTC
E  L  H  R  R  L  M  T  L  R  E  R  H  I  L  Q  Q

       160       170       180       190       200
ATCGTGAACCTTATAGAAGAAACTGGACACTTTCATATCACAAACACAAC
TAGCACTTGGAATATCTTCTTTGACCTGTGAAAGTATAGTGTTTGTGTTG
 I  V  N  L  I  E  E  T  G  H  F  H  I  T  N  T  T

       210       220       230       240       250
ATTTGATTTTGATCTTTGCTCGCTGGACAAAACCACAGTCCGTAAACTAC
TAAACTAAAACTAGAAACGAGCGACCTGTTTTGGTGTCAGGCATTTGATG
  F  D  F  D  L  C  S  L  D  K  T  T  V  R  K  L

       260       270       280       290
AGAGTTACCTGGAAACATCTGGAACATCCTGAgcggccgc
TCTCAATGGACCTTTGTAGACCTTGTAGGACTcgccggcg
Q  S  Y  L  E  T  S  G  T  S  *
```

C. Sequence of mouse *Chop* RT-PCR fragment

```
        10        20        30        40        50
gcggccgcCGTGTTCCAGAAGGAAGTGCATCTTCATACACCACCACACCT
cgccggcgGCACAAGGTCTTCCTTCACGTAGAAGTATGTGGTGGTGTGGA
 a  a  a  V  F  Q  K  E  V  H  L  H  T  P  P  H  L

        60        70        80        90       100
GAAAGCAGAACCTGGTCCACGTGCAGTCATGGCAGCTGAGTCCCTGCCTT
CTTTCGTCTTGGACCAGGTGCACGTCAGTACCGTCGACTCAGGGACGGAA
  K  A  E  P  G  P  R  A  V  M  A  A  E  S  L  P

       110       120       130       140       150
TCACCTTGGAGACGGTGTCCAGCTGGGAGCTGGAAGCCTGGTATGAGGAT
AGTGGAACCTCTGCCACAGGTCGACCCTCGACCTTCGGACCATACTCCTA
F  T  L  E  T  V  S  S  W  E  L  E  A  W  Y  E  D

       160       170       180       190       200
CTGCAGGAGGTCCTGTCCTCAGATGAAATTGGGGGCACCTATATCTCATC
GACGTCCTCCAGGACAGGAGTCTACTTTAACCCCCGTGGATATAGAGTAG
 L  Q  E  V  L  S  S  D  E  I  G  G  T  Y  I  S  S

       210       220       230       240       250
CCCAGGAAACGAAGAGGAAGAATCAAAAACCTTCACTACTCTTGACCCTG
GGGTCCTTTGCTTCTCCTTCTTAGTTTTTGGAAGTGATGAGAACTGGGAC
  P  G  N  E  E  E  E  S  K  T  F  T  T  L  D  P

       260       270       280       290       300
CGTCCCTAGCTTGGCTGACAGAGGAGCCAGGGCCAACAGAGGTCACACGC
GCAGGGATCGAACCGACTGTCTCCTCGGTCCCGGTTGTCTCCAGTGTGCG
A  S  L  A  W  L  T  E  E  P  G  P  T  E  V  T  R

       310       320       330       340       350
ACATCCCAAAGCCCTCGCTCTCCAGATTCCAGTCAGAGTTCTATGGCCCA
TGTAGGGTTTCGGGAGCGAGAGGTCTAAGGTCAGTCTCAAGATACCGGGT
 T  S  Q  S  P  R  S  P  D  S  S  Q  S  S  M  A  Q

       360       370       380       390       400
GGAGGAAGAGGAGGAAGAGCAAGGAAGAACTAGGAAACGGAAACAGAGTG
CCTCCTTCTCCTCCTTCTCGTTCCTTCTTGATCCTTTGCCTTTGTCTCAC
  E  E  E  E  E  E  Q  G  R  T  R  K  R  K  Q  S

       410       420       430       440       450
GTCAGTGCCCAGCCCGGCCTGGGAAGCAACGCATGAAGGAGAAGGAGCAG
CAGTCACGGGTCGGGCCGGACCCTTCGTTGCGTACTTCCTCTTCCTCGTC
G  Q  C  P  A  R  P  G  K  Q  R  M  K  E  K  E  Q

       460       470       480       490       500
GAGAACGAGCGGAAAGTGGCACAGCTAGCTGAAGAGAACGAGCGGCTCAA
CTCTTGCTCGCCTTTCACCGTGTCGATCGACTTCTCTTGCTCGCCGAGTT
 E  N  E  R  K  V  A  Q  L  A  E  E  N  E  R  L  K

       510       520       530       540       550
GCAGGAAATCGAGCGCCTGACCAGGGAGGTGGAGACCACACGGCGGGCTC
CGTCCTTTAGCTCGCGGACTGGTCCCTCCACCTCTGGTGTGCCGCCCGAG
  Q  E  I  E  R  L  T  R  E  V  E  T  T  R  R  A
```

```
        560       570       580       590       600
TGATCGACCGCATGGTCAGCCTGCACCAAGCATGAACAGTGGGCATCACC
ACTAGCTGGCGTACCAGTCGGACGTGGTTCGTACTTGTCACCCGTAGTGG
L  I  D  R  M  V  S  L  H  Q  A  *  T  V  G  I  T

        610       620       630       640       650
TCCTGTCTGTCTCTCCGGAAGTGTACCCAGCACCATCGCGCCAGCGCCAA
AGGACAGACAGAGAGGCCTTCACATGGGTCGTGGTAGCGCGGTCGCGGTT
 S  C  L  S  L  R  K  C  T  Q  H  H  R  A  S  A  K

        660       670       680       690       700
GCATGTGACCCTGCACTGCACTGCACATGCTGAGGAGGGGACTGAGGGTA
CGTACACTGGGACGTGACGTGACGTGTACGACTCCTCCCCTGACTCCCAT
  H  V  T  L  H  C  T  A  H  A  E  E  G  T  E  G

        710       720       730
GACCAGGAGAGGGCTCGGCTTGCACATAgcggccgc
CTGGTCCTCTCCCGAGCCGAACGTGTATcgccggcg
R  P  G  E  G  S  A  C  T  *
```

D. Sequence of human *ATF* RT-PCR fragment

```
         10        20        30        40        50
gcggccgcAAAAATTTTGAAAGACTTATCTTCTGAAGATACACGGGGCAG
cgccggcgTTTTTAAAACTTTCTGAATAGAAGACTTCTATGTGCCCCGTC
 a  a  a  K  I  L  K  D  L  S  S  E  D  T  R  G  R

         60        70        80        90       100
AAAAGGAGACGGAGAAAATTCTGGAGTTTCTGCTGCTGTCACTTCTATGT
TTTTCCTCTGCCTCTTTTAAGACCTCAAAGACGACGACAGTGAAGATACA
  K  G  D  G  E  N  S  G  V  S  A  A  V  T  S  M

        110       120       130       140       150
CTGTTCCAACTCCCATCTATCAGACTAGCAGCGGACAGTACATTGCCATT
GACAAGGTTGAGGGTAGATAGTCTGATCGTCGCCTGTCATGTAACGGTAA
S  V  P  T  P  I  Y  Q  T  S  S  G  Q  Y  I  A  I

        160       170       180       190       200
GCCCCAAATGGAGCCTTACAGTTGGCAAGTCCAGGCACAGATGGAGTACA
CGGGGTTTACCTCGGAATGTCAACCGTTCAGGTCCGTGTCTACCTCATGT
 A  P  N  G  A  L  Q  L  A  S  P  G  T  D  G  V  Q

        210       220       230       240       250
GGGACTTCAGACATTAACCATGACAAATTCAGGCAGTACTCAGCAAGGTA
CCCTGAAGTCTGTAATTGGTACTGTTTAAGTCCGTCATGAGTCGTTCCAT
  G  L  Q  T  L  T  M  T  N  S  G  S  T  Q  Q  G

        260       270       280       290       300
CAACTATTCTTCAGTATGCACAGACCTCTGATGGACAGCAGATACTTGTG
GTTGATAAGAAGTCATACGTGTCTGGAGACTACCTGTCGTCTATGAACAC
T  T  I  L  Q  Y  A  Q  T  S  D  G  Q  Q  I  L  V

        310       320       330       340       350
CCCAGCAATCAGGTGGTCGTACAAACTGCATCAGGAGATATGCAAACATA
GGGTCGTTAGTCCACCAGCATGTTTGACGTAGTCCTCTATACGTTTGTAT
 P  S  N  Q  V  V  V  Q  T  A  S  G  D  M  Q  T  Y
```

```
        360       370       380       390       400
TCAGATCCGAACTACACCTTCAGCTACTTCTCTGCCACAAACTGTGGTGA
AGTCTAGGCTTGATGTGGAAGTCGATGAAGAGACGGTGTTTGACACCACT
  Q  I  R  T  T  P  S  A  T  S  L  P  Q  T  V  V

        410       420       430       440       450
TGACATCTCCTGTGACTCTCACCTCTCAGACAACTAAGACAGATGACCCC
ACTGTAGAGGACACTGAGAGTGGAGAGTCTGTTGATTCTGTCTACTGGGG
M  T  S  P  V  T  L  T  S  Q  T  T  K  T  D  D  P

        460       470       480       490       500
CAATTGAAAAGAGAAATAAGGTTAATGAAAAACAGAGAAGCTGCTCGAGA
GTTAACTTTTCTCTTTATTCCAATTACTTTTTGTCTCTTCGACGAGCTCT
 Q  L  K  R  E  I  R  L  M  K  N  R  E  A  A  R  E

        510       520       530       540       550
ATGTCGCAGAAAGAAGAAAGAATATGTGAAATGCCTGGAAAACCGAGTTG
TACAGCGTCTTTCTTCTTTCTTATACACTTTACGGACCTTTTGGCTCAAC
  C  R  R  K  K  K  E  Y  V  K  C  L  E  N  R  V

        560       570       580       590       600
CAGTCCTGGAAAATCAAAATAAAACTCTAATAGAAGAGTTAAAAACTTTG
GTCAGGACCTTTTAGTTTTATTTTGAGATTATCTTCTCAATTTTTGAAAC
A  V  L  E  N  Q  N  K  T  L  I  E  E  L  K  T  L

        610       620       630
AAGGATCTTTATTCCAATAAAAGTGTTTGAgcggccgc
TTCCTAGAAATAAGGTTATTTTCACAAACTcgccggcg
 K  D  L  Y  S  N  K  S  V  *
```

E. Sequence of human *ERG* RT-PCR fragment

```
         10        20        30        40        50
gcggccgcAGGCAGTGGCCAGATCCAGCTTTGGCAGTTCCTCCTGGAGCT
cgccggcgTCCGTCACCGGTCTAGGTCGAAACCGTCAAGGAGGACCTCGA
 a  a  a  G  S  G  Q  I  Q  L  W  Q  F  L  L  E  L

         60        70        80        90       100
CCTGTCGGACAGCTCCAACTCCAGCTGCATCACCTGGGAAGGCACCAACG
GGACAGCCTGTCGAGGTTGAGGTCGACGTAGTGGACCCTTCCGTGGTTGC
  L  S  D  S  S  N  S  S  C  I  T  W  E  G  T  N

        110       120       130       140       150
GGGAGTTCAAGATGACGGATCCCGACGAGGTGGCCCGGCGCTGGGGAGAG
CCCTCAAGTTCTACTGCCTAGGGCTGCTCCACCGGGCCGCGACCCCTCTC
G  E  F  K  M  T  D  P  D  E  V  A  R  R  W  G  E

        160       170       180       190       200
CGGAAGAGCAAACCCAACATGAACTACGATAAGCTCAGCCGCGCCCTCCG
GCCTTCTCGTTTGGGTTGTACTTGATGCTATTCGAGTCGGCGCGGGAGGC
 R  K  S  K  P  N  M  N  Y  D  K  L  S  R  A  L  R

        210       220       230       240       250
TTACTACTATGACAAGAACATCATGACCAAGGTCCATGGGAAGCGCTACG
AATGATGATACTGTTCTTGTAGTACTGGTTCCAGGTACCCTTCGCGATGC
  Y  Y  Y  D  K  N  I  M  T  K  V  H  G  K  R  Y
```

```
       260       270       280       290       300
CCTACAAGTTCGACTTCCACGGGATCGCCCAGGCCCTCCAGCCCCACCCC
GGATGTTCAAGCTGAAGGTGCCCTAGCGGGTCCGGGAGGTCGGGGTGGGG
A  Y  K  F  D  F  H  G  I  A  Q  A  L  Q  P  H  P

       310       320       330       340       350
CCGGAGTCATCTCTGTACAAGTACCCCTCAGACCTCCCGTACATGGGCTC
GGCCTCAGTAGAGACATGTTCATGGGGAGTCTGGAGGGCATGTACCCGAG
 P  E  S  S  L  Y  K  Y  P  S  D  L  P  Y  M  G  S

       360       370       380       390       400
CTATCACGCCCACCCACAGAAGATGAACTTTGTGGCGCCCCACCCTCCAG
GATAGTGCGGGTGGGTGTCTTCTACTTGAAACACCGCGGGGTGGGAGGTC
  Y  H  A  H  P  Q  K  M  N  F  V  A  P  H  P  P

       410       420       430       440       450
CCCTCCCCGTGACATCTTCCAGTTTTTTTGCTGCCCCAAACCCATACTGG
GGGAGGGGCACTGTAGAAGGTCAAAAAAACGACGGGGTTTGGGTATGACC
A  L  P  V  T  S  S  S  F  F  A  A  P  N  P  Y  W

       460       470       480       490       500
AATTCACCAACTGGGGGTATATACCCCAACACTAGGCTCCCCACCAGCCA
TTAAGTGGTTGACCCCCATATATGGGGTTGTGATCCGAGGGGTGGTCGGT
 N  S  P  T  G  G  I  Y  P  N  T  R  L  P  T  S  H

       510       520       530       540
TATGCCTTCTCATCTGGGCACTTACTACTAAgcggccgc
ATACGGAAGAGTAGACCCGTGAATGATGATTcgccggcg
  M  P  S  H  L  G  T  Y  Y  *
```

F. Sequence of *Ews-FliI* junction in pC2A-neo

Ews exon 7

```
ACTAGTTACCCCCCTCAGACTGGATCC
 T  S  Y  P  P  Q  T  G  S
```

Ews exon 7 knock-in with *FliI* in pC2A

```
                     BamHI/BglII NotI
ACTAGTTACCCCCCTCAGACTGGATCTGCGGCCGCAGACCCTTCTTATGAC
 T  S  Y  P  P  Q  T  G  S  a  a  a  D  P  S  Y  D
```

G. Sequence of *Ews-ERG* mRNA junction

```
             Ews                                              ERG
CAACAGAGCAGCAGCTACGGGCAGCAGAGGGGATCTGCGGCCGCAGGCAGTGGCCAGATCCAGCTTTGGCA
G
 Q  Q  S  S  S  Y  G  Q  Q  r  g  s  a  a  a  G  S  G  Q  I  Q  L  W  Q
```

H. Genomic restriction map of the *Ews* gene around exon 7 indicating sites of knock-in homologous recombination

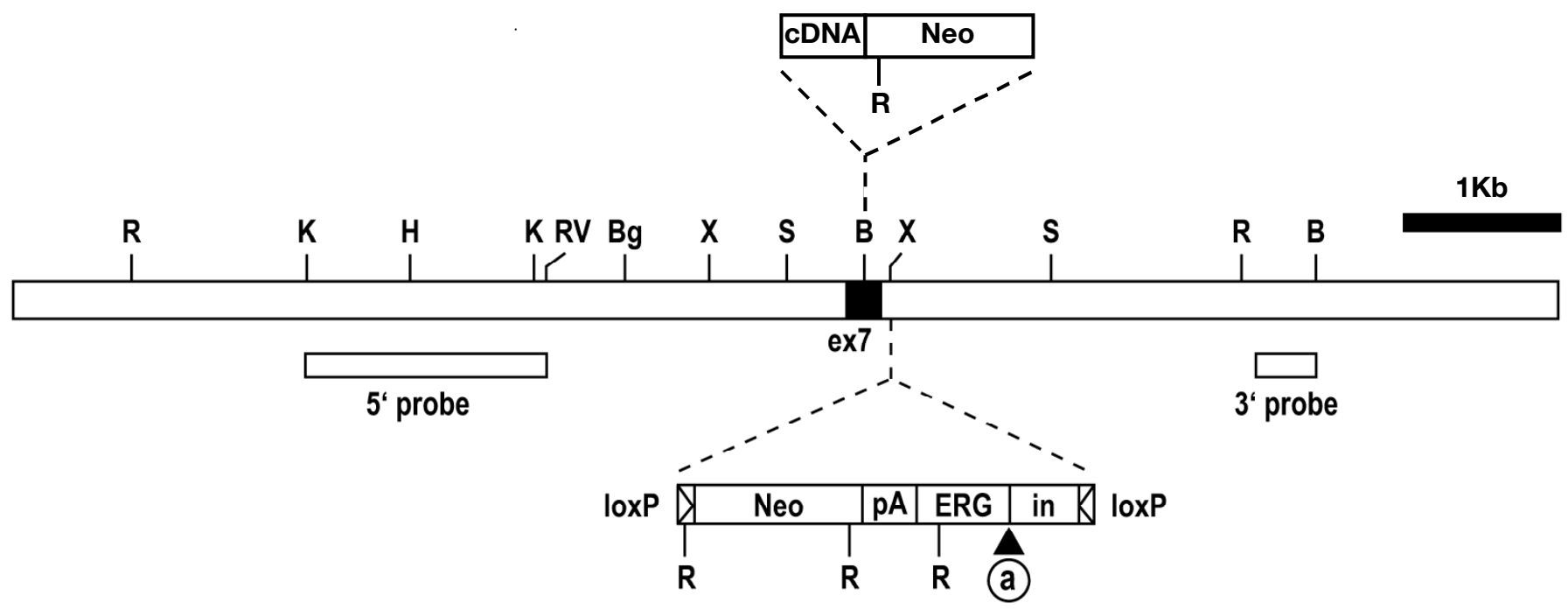

Sequences of fusion cDNA for generation of *Ews* knock-in or invertor targeting clones

A-E. Each cDNA sequence was made by RT-PCR of the relevant mRNA sequence usually involved in human translocations. cDNA sequences were cloned as NotI PCR fragments into pC2A-neo [1] for generation of targeted knock-in *Ews* genes in ES cells. *Fli1* cDNA was amplified from MEL RNA (**A**), *AF9* cDNA from HEL RNA (**B**), *Chop* from 3T3L1 RNA (**C**), *ATF1* from Jurkatt RNA (**D**) and *ERG* from COLO320 RNA (**E**) (latter was used for the invertor cassette). The NotI restriction sites (lower case) at each end of the PCR fragments were added as part of the PCR primers, for cloning into the NotI site of pC2A-neo [1]. The pC2A-neo cassettes with each added cDNA sequence was cleaved with BglII and the fragment (which includes the MC1-neo-pA fragment for selection of targeted clones) cloned into the BamHI site of *Ews* exon 7, to generate in-frame fusions between the *Ews* coding region and the incoming cDNA. The invertor cassette included a short segment of intronic sequence (from intron 4 of the mouse *Af4* gene) including the acceptor splice site, the *ERG* cDNA, a polyA site and the MC1-neo-pA segment for selection of homologous recombinant clones, all flanked by *loxP* sites with an orientation compatible for inversional recombination by Cre.

F. The fusion junction of *Ews* exon 7 BamHI site and the BamHI/BglII junction of *Ews* and *Fli1* (as a knock-in example; similar sequences exist of the *Chop* and *ATF1* junctions).

G. The junctional sequence of the *Ews-ERG* mRNA from spleen of an *Ews-Erg; Rag-Cre* invertor mouse. The underlined NotI restriction enzyme recognition site derives from the pC2A-neo transfer vector [1].

All the derived protein sequences are shown as single letter code.

H. Restriction map of part of the *Ews* gene showing the site for knock-in, by gene targeting, of *Ews* fusion partners (BamHI site in exon 7) and *ERG* invertor cassette (XbaI site 3' of exon 7).

References

1. Forster A, Pannell R, Drynan LF, Codrington R, Daser A, et al. (2005) The invertor knock-in conditional chromosomal translocation mimic. Nature Methods 2; 27-30
