# Supplementary material for: The Ews-ERG Fusion Protein Can Initiate Neoplasia from Lineage-Committed Haematopoietic Cells
Source: PLoS Biol. 2005 Jun 28;3(8):e242. doi: 10.1371/journal.pbio.0030242 (PMC1159166; doi:10.1371/journal.pbio.0030242)
Supplement: Supplementary file 2 [file pbio.0030242.sg002.pdf]

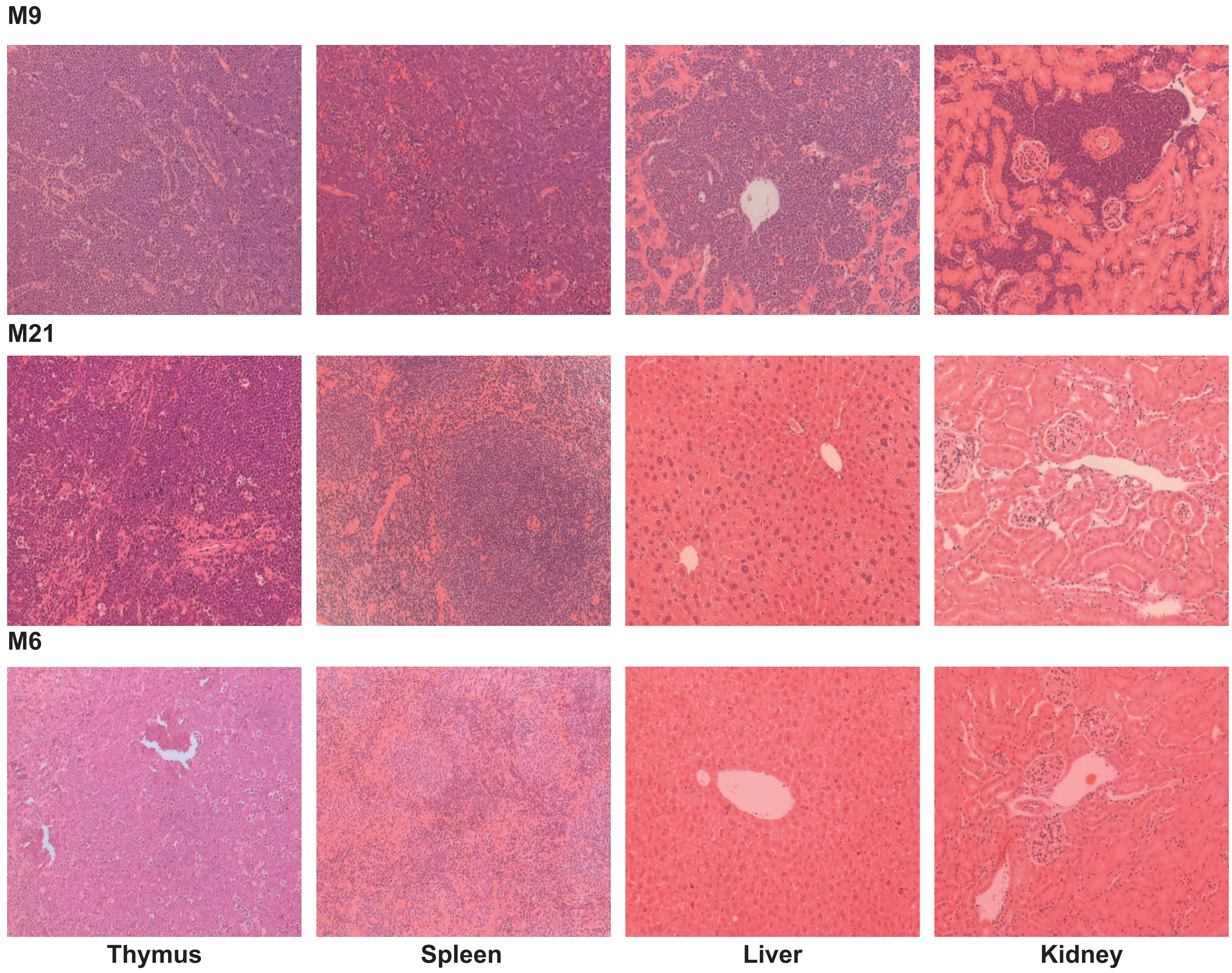
M9
M21
M6
Thymus
Spleen
Liver
Kidney

Supplemental Figure S2

Histological characteristics of leukaemias in *Ews-ERG* invertor mice

Ews-ERG;Rag1-Cre mice (and *Ews-ERG* controls) were monitored for approximately 17 months. Individual mice were culled and post-mortem conducted when signs of ill health were observed. Leukaemia was established by various criteria (see Methods). Histological sections of tissues from mice with thymoma, illustrating three levels of severity of leukaemic infiltration. Tissues were fixed in 10% formalin and wax embedded, followed by sectioning and staining with H&E. Mouse M9 had the most severe phenotype, with thymus and spleen heavily disrupted by tumour cells and marked peri-vascular deposits of leukocytes in liver and kidney. Mouse M21 had severe alterations to thymus and spleen but little involvement of liver or kidney. M6 has the least severe phenotype, with only its thymus being involved with an abnormal structure.

Sections were 0.4μm thick and photographed at 400X times.
