# Supplementary material for: The Ews-ERG Fusion Protein Can Initiate Neoplasia from Lineage-Committed Haematopoietic Cells
Source: PLoS Biol. 2005 Jun 28;3(8):e242. doi: 10.1371/journal.pbio.0030242 (PMC1159166; doi:10.1371/journal.pbio.0030242)
Supplement: Supplementary file 3 [file pbio.0030242.sg003.pdf]

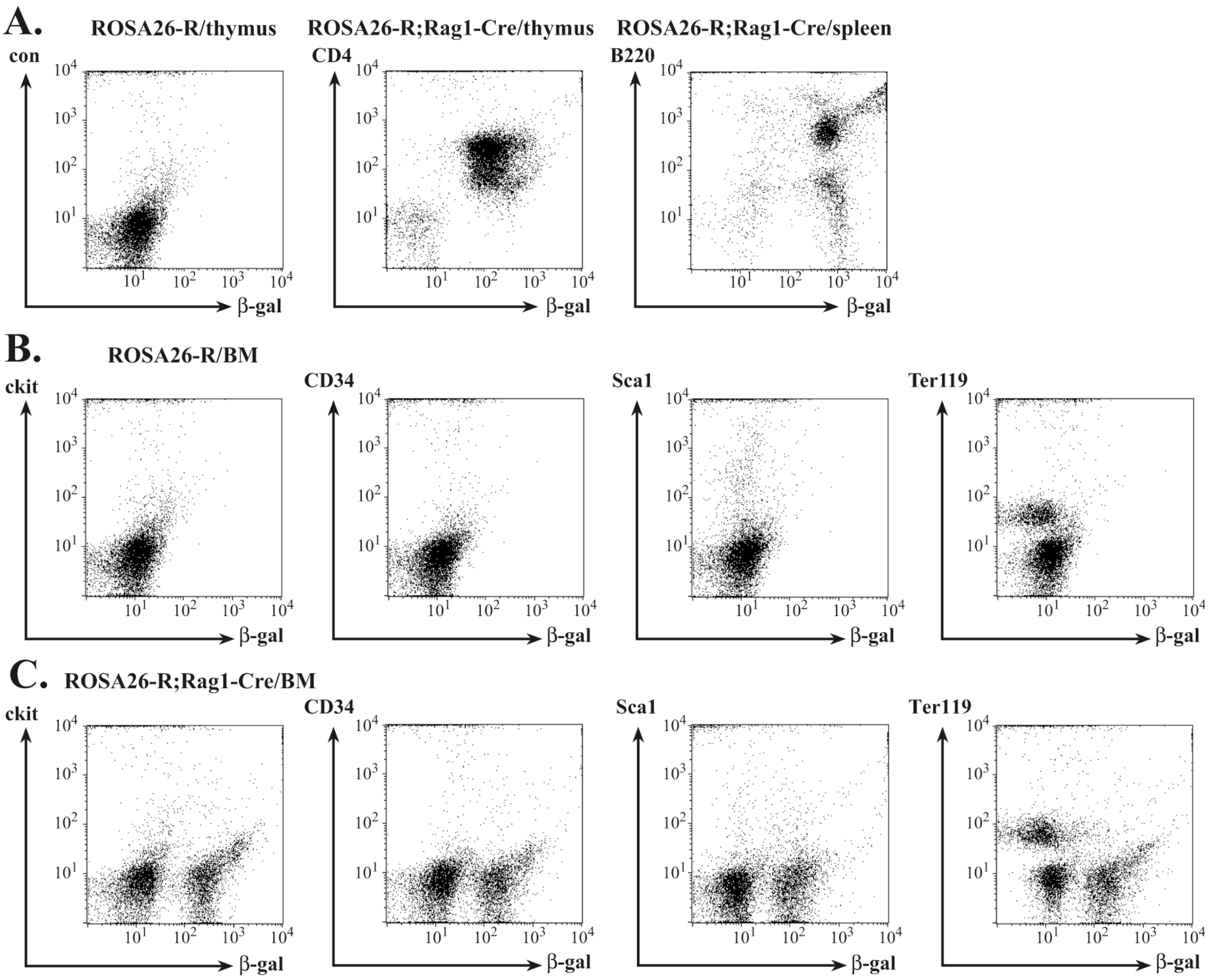
A.
ROSA26-R/thymus
ROSA26-R;Rag1-Cre/thymus
ROSA26-R;Rag1-Cre/spleen
con
CD4
B220
β-gal
B.
ROSA26-R/BM
ckit
CD34
Sca1
Ter119
C.
ROSA26-R;Rag1-Cre/BM
10^1
10^2
10^3
10^4

Supplemental Figure S3

ROSA-βgal reporter assay for expression of *Rag1-Cre* allele

ROSA-lacZ reporter mice (ROSA26-R) [1] carry a *lacZ* gene linked to a *loxP*-polyadenylation stop. Removal of this loxP-polyadenylation stop segment activates the *lacZ* gene under the control of the ROSA promoter. *ROSA-lacZ* reporter mice were crossed with *Rag1-Cre* mice [2] and at 3 months of age, single cell suspensions were prepared from thymus, spleen and bone marrow. Thymus (A), spleen (A), bone marrow (BM) (B, C) cells were incubated with PE-conjugated antibodies (BD-Pharmingen, as indicated in the figure) and with the β-galactosidase substrate FDG (Molecular Probes), after permeabilisation of the cells according to Manufacturer's instructions, yielding a green fluorescent product. FACS analysis was conducted using a FACSCalibur with fluorescent antibodies (red, y axis) and b-gal (green, x axis) and data were analyzed with CellQuest software.

References

1. Soriano P (1999) Generalized lacZ expression with the ROSA26 Cre reporter strain. Nat Genet 21: 70-71.
2. McCormack MP, Forster A, Drynan LF, Pannell R, Rabbitts TH (2003) The *LMO2* T-cell oncogene is activated via chromosomal translocations or retroviral insertion during gene therapy but has no mandatory role in normal T-cell development. Mol Cell Biol 23: 9003-9013.
