# Supplementary material for: The Ews-ERG Fusion Protein Can Initiate Neoplasia from Lineage-Committed Haematopoietic Cells
Source: PLoS Biol. 2005 Jun 28;3(8):e242. doi: 10.1371/journal.pbio.0030242 (PMC1159166; doi:10.1371/journal.pbio.0030242)
Supplement: Supplementary file 4 [file pbio.0030242.st001.doc]

## Supplemental Table S1

A

| **Targeting vector** | **Colonies analyzed** | **No. of targeted clones** |
| --- | --- | --- |
| *Ews-cassette control* | 64 | 4 |
| *Ews-AF9* | 95 | 2 |
| *Ews-Fli1* | 170 | 0 |
| *Ews-Chop* | 144 | 0 |
| *Ews-ATF1* | 200 | 0 |

B

| **Targeted ES cell** | **Blastocyst nos. transferred** | **No. of chimeras** |
| --- | --- | --- |
| *Ews-cassette control* | 143 | 18 |
| *Ews-AF9* | 78 | 20 |

Frequency of targeted clones and chimaera generation with *Ews* knock-in ES clones

*Ews* knock-in targeted clones were made using homologous recombination in ES (CCB) cells and successful targeting was established by growth on G418 (due to expression of the *neo* resistance gene) and gancyclovir (homologous events deleting the *Hsv-tk* gene from the targeting vector).

**A.** The number of targeted clones identified from the indicated number of colonies analyzed. *Ews* cassette control represents the knock-in of pC2A-neo [1] lacking additional cDNA sequences, into *Ews* exon 7. *Ews-AF9, Fli1, Chop* and *ATF1* represent respectively knock-ins of fusion cDNAs as shown in Supplemental Figure S1.

B. The number of chimaeras (identified by coat color) resulting from injection of *Ews*-targeted ES clones into blastocysts following transfer to recipient females.

##### References

1. Forster A, Pannell R, Drynan LF, Codrington R, Daser A, et al. (2005) The invertor knock-in conditional chromosomal translocation mimic. Nature Methods 2: 27-30
